# Odorranalectin Is a Small Peptide Lectin with Potential for Drug Delivery and Targeting

**DOI:** 10.1371/journal.pone.0002381

**Published:** 2008-06-11

**Authors:** Jianxu Li, Hongbing Wu, Jing Hong, Xueqing Xu, Hailong Yang, Bingxian Wu, Yipeng Wang, Jianhua Zhu, Ren Lai, Xinguo Jiang, Donghai Lin, Mark C. Prescott, Huw H. Rees

**Affiliations:** 1 Biotoxin Units of Key Laboratory of Animal Models and Human Disease Mechanisms, Kunming Institute of Zoology, Chinese Academy of Sciences, Kunming Yunnan, China; 2 School of Pharmacy, Fudan University, Shanghai, China; 3 Shanghai Institute of Materia Medica, Chinese Academy of Sciences, Shanghai, China; 4 School of Biological Sciences University of Liverpool, Liverpool, United Kingdom; 5 Graduate School of the Chinese Academy of Sciences, Beijing, China; Center for Genomic Regulation, Spain

## Abstract

**Background:**

Lectins are sugar-binding proteins that specifically recognize sugar complexes. Based on the specificity of protein–sugar interactions, different lectins could be used as carrier molecules to target drugs specifically to different cells which express different glycan arrays. In spite of lectin's interesting biological potential for drug targeting and delivery, a potential disadvantage of natural lectins may be large size molecules that results in immunogenicity and toxicity. Smaller peptides which can mimic the function of lectins are promising candidates for drug targeting.

**Principal Findings:**

Small peptide with lectin-like behavior was screened from amphibian skin secretions and its structure and function were studied by NMR, NMR-titration, SPR and mutant analysis. A lectin-like peptide named odorranalectin was identified from skin secretions of *Odorrana grahami*. It was composed of 17 aa with a sequence of YASPKCFRYPNGVLACT. L-fucose could specifically inhibit the haemagglutination induced by odorranalectin. ^125^I-odorranalectin was stable in mice plasma. In experimental mouse models, odorranalectin was proved to mainly conjugate to liver, spleen and lung after i.v. administration. Odorranalectin showed extremely low toxicity and immunogenicity in mice. The small size and single disulfide bridge of odorranalectin make it easy to manipulate for developing as a drug targeting system. The cyclic peptide of odorranalectin disclosed by solution NMR study adopts a β-turn conformation stabilized by one intramolecular disulfide bond between Cys6-Cys16 and three hydrogen bonds between Phe7-Ala15, Tyr9-Val13, Tyr9-Gly12. Residues K5, C6, F7, C16 and T17 consist of the binding site of L-fucose on odorranalectin determined by NMR titration and mutant analysis. The structure of odorranalectin in bound form is more stable than in free form.

**Conclusion:**

These findings identify the smallest lectin so far, and show the application potential of odorranalectin for drug delivery and targeting. It also disclosed a new strategy of amphibian anti-infection.

## Introduction

The biological barriers between the site of drug action and drug delivery are becoming increasingly important for the development of effective and safe medicines as mentioned by Lehr et al and Bies et al [1], [2]. Being proteins or glycoproteins themselves, lectins bind to sugar-moieties of the cell membrane. Typically involving a high number of binding sites and determined by a specific sugar code, lectin binding is usually rapid and strong. Lectins have been focused upon for more than 20 years and are becoming excellent candidates for drug delivery and targeting [3], [4]. Some of problems are associated with lectins because of their large molecular weights. Molecular weights of most lectins are more than 10 KDa that likely results in toxicity and immunogenicty [2], [5]. These problems are probably overcome by small size lectins with high target specificity. As far as we know, two small size lectins have been found. They are *Selenocosmia huwena* lectin-I [6] and θ-defensin [7]. *Selenocosmia huwena* lectin-I is identified from the venom of the Chinese bird spider *Selenocosmia huwena*. It is composed of 32 residues including three disulfide bridges with homology with N-terminal fragment of great nettle lectin. θ-defensin is purified from the leukocytes and bone marrow of the rhesus macaque (*Macaca mulatta*). θ-defensin is an antimicrobial peptide with capability to protect cells from *in vitro* infection by HIV-1. θ-defensin is circular, tetracyclic peptides with three disulfide bridges connecting its antiparallel β-sheets and composed of 18 residues. It can specially bind to galactosylceramide [7]. Although they have a small size, it is not easy to manipulate and develop *Selenocosmia huwena* lectin-I and θ-defensin as drug targeting systems because of their complex circular structures and multiple disulfide bridges.

## Results

### The smallest lectin was identified from skin secretions of the frog, *O. grahami*

**Figure 1:**
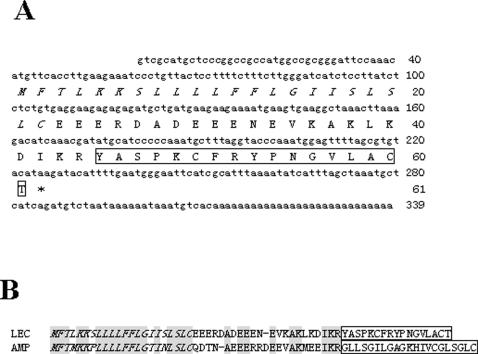
cDNA sequence of odorranalectin, (A) cDNA nucleotide sequence and deduced amino acid sequence of odorranalectin precursor. The amino sequence of mature odorranalectin is boxed. The stop codon is indicated by a star (*); (B) The precursor of odorranalectin (LEC) shares a similar signal peptide with the antimicrobial peptide Nigrocin-OG20 (AMP) isolated from *O. grahami* [11]. Both of the mature peptides are preceded by a Lys-Arg motif, which is a typical processing site for endoproteolytic cleavage. The signal peptide is given in italics. The mature peptide is boxed. Conserved amino acid residues are shaded.

We purified and characterized a small peptide named odorranalectin with lectin-like activity from skin secretions of the frog, *O. grahami* (Fig. S1). It was composed of 17 amino acid residues with a sequence of YASPKCFRYPNGVLACT containing a single disulfide bridge (Fig. 1). L-fucose could specifically inhibit the haemagglutination induced by odorranalectin. Odorranalectin is the smallest known lectin. Several cDNA clones encoding precursors of odorranalectin were cloned from a skin cDNA library of *O. grahami*. It was found to have an open reading frame that encodes a polypeptide composed of 61 amino acids, including the mature odorranalectin sequence (Fig. 1A, GenBank accession EU072454). The structural organization of the precursor is quite similar to amphibian antimicrobial peptide precursors, comprising a signal peptide sequence, an N-terminal spacer peptide region containing several aspartic and glutamic acid residues, and the mature peptide at the C-terminus of the precursor. There is a di-basic cutting site (-K^42^R^43^-) for trypsin-like proteases between spacer peptide and mature peptide. The amino acid sequences deduced from the cDNA sequences match well with the amino acid sequences determined by Edman degradation. Since the precursor of odorranalectin shares a similar signal and propiece peptide with the previously identified amphibian antimicrobial peptides [8], [9] (Fig. 1B), we suspected that it had antimicrobial activities. In contrast to our speculation, odorranalectin had no antimicrobial activities (data not shown).

### Microbe-agglutinating and hemagglutinating activity of odorranalectin

**Figure 2:**
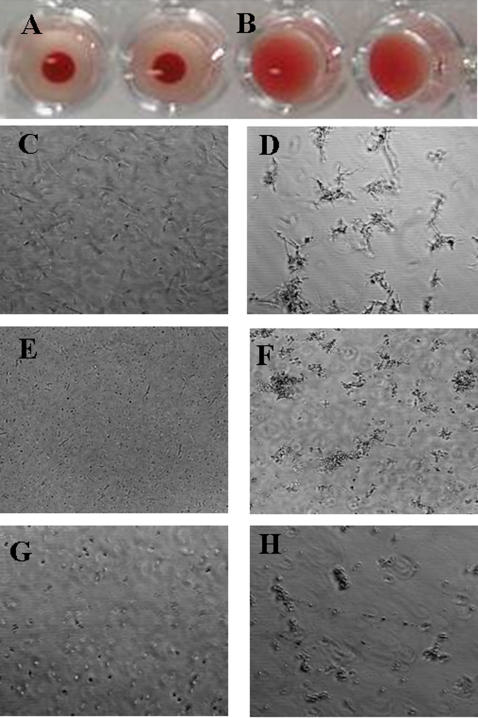
Hemagglutinating and microbe-agglutinating activities of odorranalectin. odorranalectin could agglutinate rabbit erythrocytes. In control (A) wells no lectins were added; in sample (B) wells 4 hemagglutination doses of odorranalectin were added; 4 hemagglutination doses of odorranalectin could also agglutinate microorganisms as illustrated in (C), untreated *E.coli* DH5α; (D), odorranalectin treated *E.coli* DH5α; (E), untreated *C. albicans*; (F), odorranalectin treated *C. albicans;* (G), untreated *S. aureus*; (H), odorranalectin treated *S. aureus*.

Odorranalectin could strongly agglutinate intact, trypsin-, or formaldehyde-treated rabbit erythrocytes (Fig. 2B; Table S1), however more odorranalectin was needed for agglutinating proteinase-treated rabbit erythrocytes. The minimum concentration (MIC) to agglutinate intact human erythrocytes is 0.75 µg/ml (Table S1). EDTA treatment and metal cation addition to odorranalectin did not affect the agglutinating activity, which suggested that odorranalectin did not depend on metal cation to exert its lectin-like activity. Considering the similarity of the overall structure of odorranalectin precursor with amphibian antimicrobial peptides, some biological activities including microbe-killing, microbe-agglutinating, and histamine-releasing, which are related to innate immunity have been tested. Although odorranalectin had no direct microbe-killing ability, it could agglutinate microorganisms and induce histamine release from mast cells (Fig. 2C–2H; Table S2). Among the tested microorganisms, the minimal concentration of odorranalectin to agglutinate *Escherichia coli DH5a* (Fig. 2D), *Candida albicans* (*ATCC2002*) (Fig. 2F), and *Staphylococcus aureus* (*ATCC2592*) (Fig. 2H) is 0.625, 1.25, and 2.50 µg/ml, respectively (Table S2). *E. coli DH5a* was most sensitive to odorranalectin. Furthermore, odorranalectin had moderate histamine-releasing ability, 25 and 50 µg/ml odorranalectin could induce 5.72% and 12.48% histamine release, respectively (Table S3). Some lectins such as mannose-binding lectin (MBL) [10]–[12] distributed in serum can recognize invading microbes by binding to surface mannose residues or to peptidoglycan. This binding triggers the lectin-activated complement pathway, which is initiated by recruitment of the serine proteases MASP1 and via interactions with the MBL collagenous domain. Odorranalectin has no collagenous domain. Our experiments also demonstrated that odorrananlectin had no effect on the lectin-activated complement pathway (data not shown). Synergy between antimicrobial peptides has been reported [9], [13], [14]. We conducted a study to explore the synergy between the amphibian lectin and amphibian antimicrobial peptides Nigrocin-OG13, Nigrocin-OG21 from *O. grahami* skin. Unexpectedly, while individually these peptides had no effect on HIV, in combination they did, although with low selectivity (unpublished data). All the results suggested that odorranalectin could exert some functions related to innate immunity and probably take part in anti-infection defence of amphibian.

### Carbohydrate-binding specificity of odorranalectin

**Figure 3:**
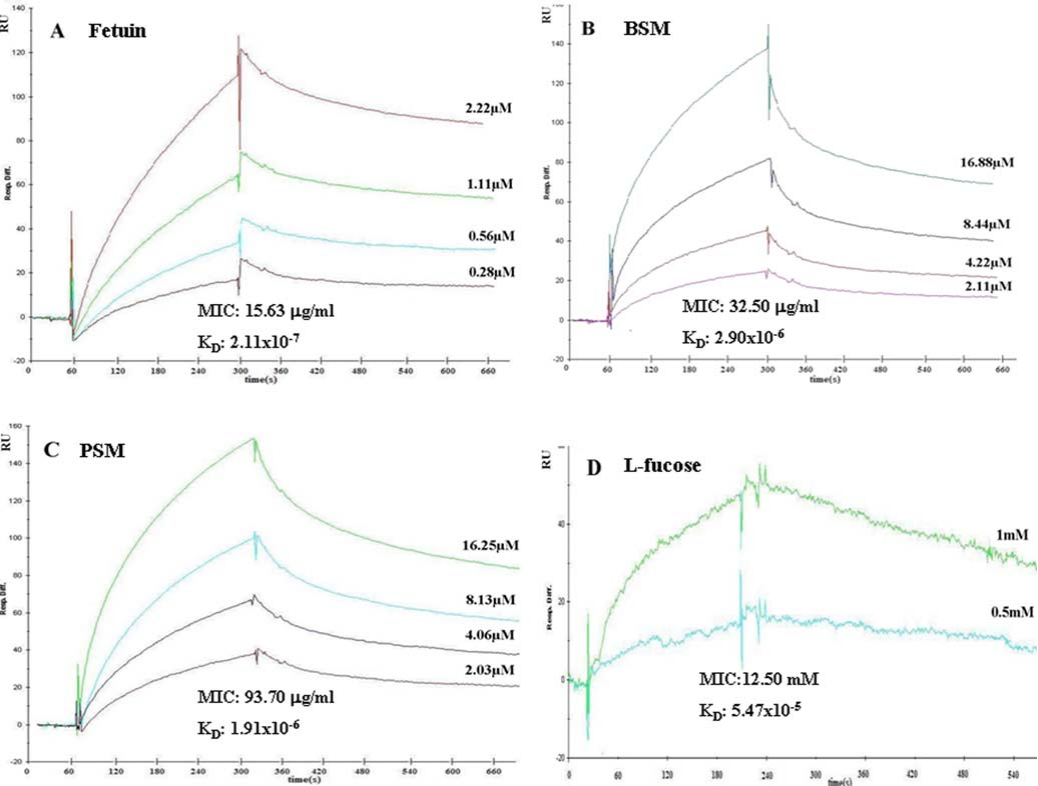
Sensor-grams showing the interaction between immobilized odorranalectin and glycoproteins or sugar with different concentrations as indicated. (A), fetuin; (B), bovine submaxillary mucin (BSM); (C), porcine stomach mucin (PSM); (D), L-fucose; *RU,* resonance units; MIC is the minimum inhibitory concentrations required for inhibition of four hemagglutination doses of odorranalectin. sucrose, D(+)-cellobiose, D(+)-melezitose monohydrate, inulin, D(+)-raffinose pentahydrate, D(+)-galacturonic acid, lactose, D-glucose,D-ribose, D-trehalose, isopropy-β-D-thiogalactoside, 2-nitrophenyl-β-D-galactopyranoside, dulcitol, L-sorbose,D-sorbose, D-froctose, L(+)-rhamnose monohydrate, D(+)-xylose, D(+)-galactose, D(+)-arabinose, N-acetylgalactosamine, N-acetylglucosamine N-acetylneuraminic acid, D-sorbitol, inositol, adonitol, D-mannose, N-acetyl-D-mannosamine, 5-bromo-4-chloro-3-indoxyl-β-D-galactopyranoside, N-glycolylneuraminc acid, D(+)-maltose monohydrate, and heparin did not inhibit at all at concentrations up to 200 mM. Association and dissociation rate constants (*ka* and *kd*) were calculated by using BIA evaluation 4.0 software (Biacore AB, Sweden). The affinity constant (*K_D_*) was calculated from the *ka* and *kd*. For the calculation of rate constants, samples were appropriately diluted in 10 mM HEPES-buffered saline containing 3 mM EDTA and 0.005% surfactant P-20, pH 7.4 (HBS-EP) at various concentrations.

Carbohydrate-binding specificity of odorranalectin was examined by hemagglutination-inhibition test. Among the 33 tested monomeric sugars, only L-fucose could inhibit its hemagglutinating and microbe-agglutinating activities (Fig. 3; Table S2). It is suggested that L-fucose could be the ligand of odorranalectin. Surface plasmon resonance (SPR) analysis further demonstrated that L-fucose could specifically bind to odorranalectin with a binding affinity (*K*_D_) of 5.47×10^−5^ M (Fig. 3D; Table S4). The mucin-binding specificity of odorranalectin was also investigated by SPR analyses (Fig. 3A–3C; Table S4). Odorranalectin was immobilized on the sensor chip CM-5 by amine coupling and four glycoproteins: Bovine submaxillary mucin (BSM), porcine stomach mucin (PSM), fetuin, and transferrin were used as analytes. All the glycoproteins except transferrin could inhibit the hemagglutinating activity of odorranalectin, among which, fetuin was the most effective inhibitor. The sensorgrams and the kinetic data of the binding are shown in Fig. 3 and Table S4, respectively. Transferrin did not bind to the immobilized odorranalectin. The binding of all the other glycoproteins and L-fucose to the immobilized lectin fitted best to a 1∶1 binding model among the various models in the evaluating software. Among the tested glycoproteins, the affinity of fetuin was the strongest (*K*_D_ = 2.11×10^−7^ M) among the analytes and that of BSM was the weakest (*K*_D_ = 2.9×10^−6^ M). Odorranalectin binding to monomeric sugar and glycoproteins did not require calcium. Taken together, these results suggested that odorranalectin is a pattern-recognition peptide that recognizes the microbe-associated molecular pattern represented by the extended glycan chains of peptidoglycan. The interaction between odorranalectin and L-fucose was further demonstrated in the NMR titration experiments.

### NMR titration

**Figure 4:**
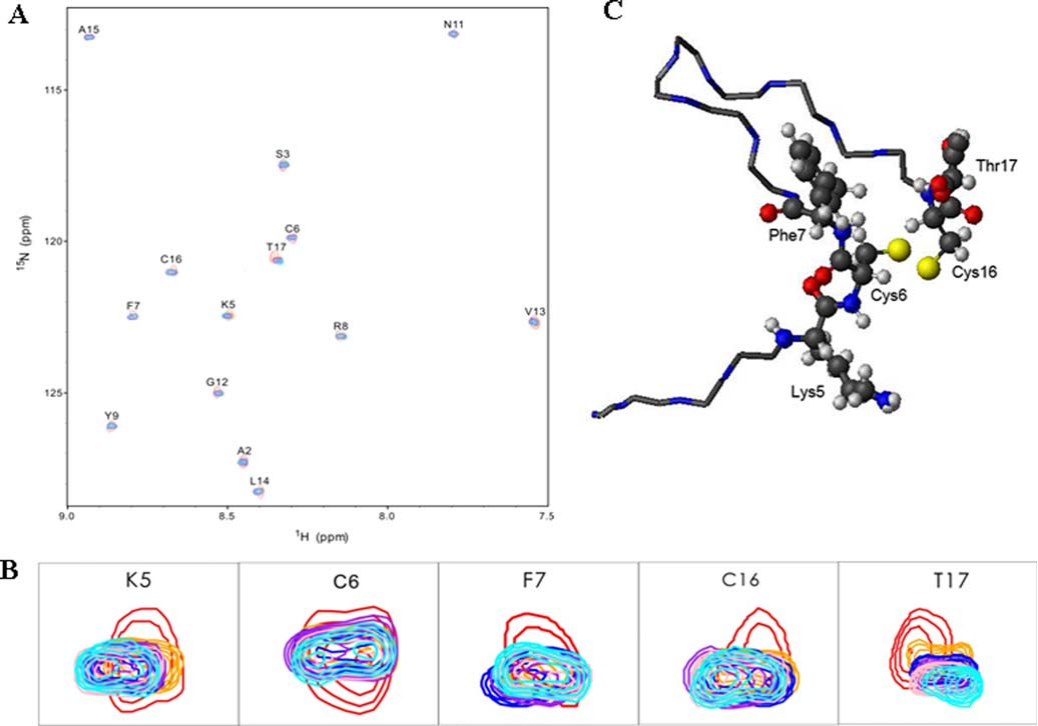
Titration of L-fucose to odorranalectin. (A) Superposition of the ^1^H-^15^N HSQC spectra of odorranalectin free and in the complex with L-fucose. The molar ratios of odorranalectin to L-fucose were 1∶0 (red), 1∶0.25 (orange), 1∶0.5 (blue), 1∶0.75 (purple), 1∶2 (pink), 1∶3 (cyan), respectively. (B) Each of five isolated peaks was split into two peaks upon titration with L-fucose which was indicative of interaction between odorranalectin and L-fucose. (C) Five residues consist of the L-fucose binding site on odorranalectin, which were displayed with side-chains in the structure of odorranalectin. Atoms C, O, N, H, S were shown in black, red, blue, light gray and yellow, respectively.

To understand the structure-function relation of odorranalectin, we studied the interaction between odorranalectin and L-fucose using NMR titration experiments. ^1^H-^15^N HSQC spectra were used to monitor peak changes during the L-fucose titration process and to map the binding site of L-fucose on odorranalectin. Superposition of the ^1^H-^15^N HSQC spectra of odorranalectin free and in the complex with L-fucose was shown in Fig. 4 A. It was observed that each of five isolated peaks related to residues K5, C6, F7, C16 and T17, respectively, was spilt into two peaks upon titration with L-fucose, shown in Fig. 4B, while other peaks did not experience significant changes. This observation indicated that odorranalectin was bound to L-fucose. Residues K5, C6, F7, C16 and T17 consist of the binding site of L-fucose on odorranalectin, displayed in Fig. 4C. It was further confirmed by mutational analyses. Most of mutated peptides lost the ability to bind with L-fucose as shown in table S5. Furthermore, most peaks became more or less stronger upon titration with L-fucose, which may indicate that the structure of odorranalectin in bound form is more stable than in free form. It was suggest that the L-fucose binding may increase the rigidity of odorranalectin.

### Stability of ^125^I-odorranalectin *in vitro* and mouse tissue distribution of ^125^I-odorranalectin *in vivo*

**Figure 5:**
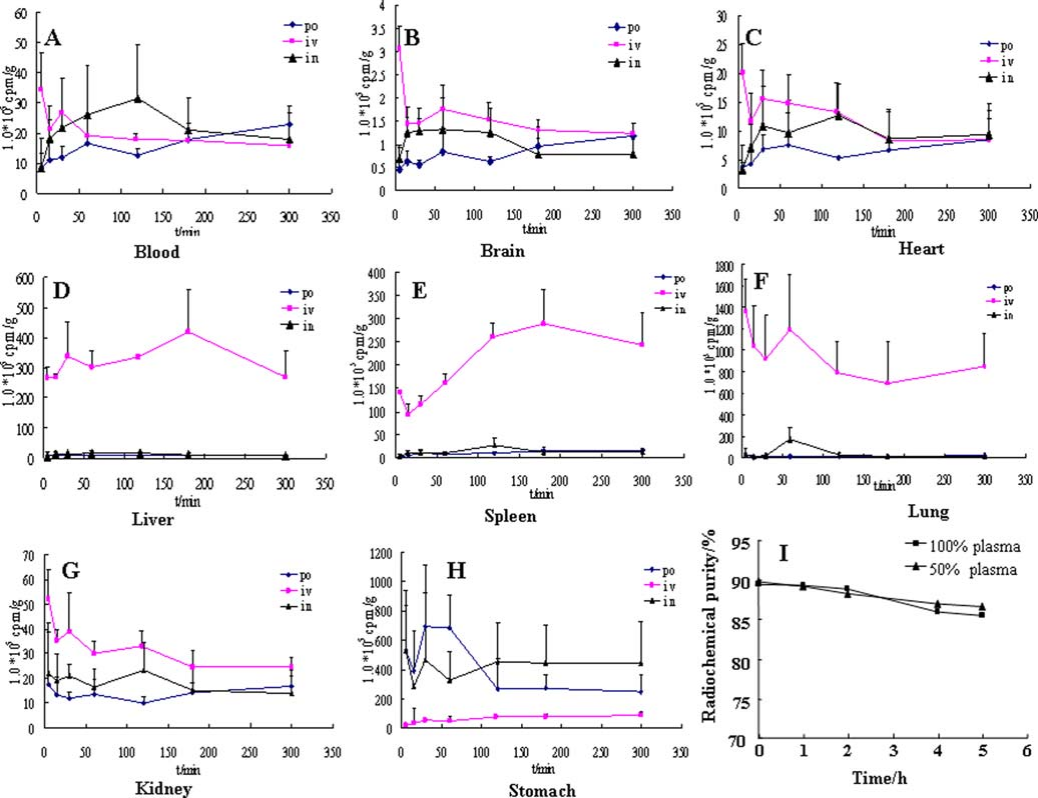
Radiocounting-time curves of blood and different tissues after per oral (Rhombus), intravenous (Rectangular) and intranasal (Triangle) administration into mice (n = 3), and the stability of ^125^I-odorranalectin in plasma.

^125^I is often used to label protein or polypeptide because it can substitute a hydrogen atom in the tyrosine and has suitable radioactive half-life (about 60 days). In this study, odorranalectin was labeled by ^125^I and purified to 99% by HPLC. Purified ^125^I-odorranalectin was used to study stability in plasma and tissue distribution of mice *in vivo*. Fig. 5I indicated that ^125^I-odorranalectin could exist stably in the mice plasma for at least 5 hours, and the key point of *in vitro* test witnessed that ^125^I isotope tracer method was suitable for further tagging investigation *in vivo*. Different lectins have different carbohydrate specificities [15], and distribution of various carbohydrates in mucous membranes is also different. But through different routes of administration, such as per oral, intravenous, and per nasal administration routes of ^125^I-odorranalectin at an equal radiation dose, the specificity of its glycosyl-binding may be identified. Radiocounting-time curves of ^125^I-odorranalectin in different tissues were plotted (Fig. 5A–5H), and total accumulation of radiocounting (AUC_0→t_) in each tissue were shown in Table S6 and Table S7. The AUC values of liver, spleen and lung were much larger than other tissues after intravenous administration; this striking difference might be caused by two reasons: 1), ^125^I-odorranalectin could be specifically conjugated to liver, spleen and lung; 2), the aggregates of ^125^I-odorranalectin with erythrocyte were phagocytosed by phagocytic cells abundant in liver and spleen, and the firs-pass effect of lung. Compared to intravenous administration, however, the amounts of ^125^I-odorranalectin absorbed into blood circulation after oral and nasal administration, seemed to no distinctions, and the amounts through intranasal administration were even more than that of intravenous, while the uptakes of liver, spleen and lung were striking much less than those of intravenous injection, the possible reason was that the radioactivity determined in the blood after per oral or intranasal administration was not caused by ^125^I-odorranalectin. According to the AUC_stomach_ values, it was not difficult to understand that the bulk of ^125^I-odorranalectin was swallowed when per nasal administration. On the basis of the results above, ^125^I-odorranalectin might be degraded or lost its activity when absorbing through gastrointestinal tract after per oral and nasal administration.

### Immunogenicity and toxicity of odorranalectin

To assess the immunogenicity of odorranalectin, groups of mice (n = 10, seven-week-old, female) were immunized by odorranalectin and other control proteins (ovalbumin, OVA, a poorly immunogenic protein; wheat germ agglutinin, WGA), using 0.1 M sodium bicarbonate as blank control. On days 1, 14, and 35, 100 µg OVA or odorranalectin in sterile 0.1 M sodium bicarbonate was intravenously administered. After three immunizations with OVA, WGA, odorranalectin, and 0.1 M sodium bicarbonate, the detection of specific antibodies in sera was performed by enzyme-linked immunosorbent assay (ELISA). The maximum specific IgG titre for OVA, WGA, and odorranalectin was 1∶200, 1∶1600, and 1∶200, respectively, and the maximum specific IgA titre for OVA, WGA, and odorranalectin was 1∶300, 1∶2000, and 1∶200, respectively. Odorranalectin had similar immunogenicity with OVA, which is a kind of poorly immunogenic protein. Compared with another lectin (WGA), odorranalectin had extremely low immunogenicity. Single dose acute toxicity studies (24 h) in animals are necessary for pharmaceuticals intended for humans. Odorranalectin was tested for acute toxicity by i.v. delivery in female outbred mice. The maximum tolerable dose of odorranalectin by i.v. delivery was between 75 and 100 mg/kg. It has been mentioned that odorranalectin had no effect on complement activation. All the results suggested that odorranalectin had extremely low toxicity.

### Solution structure of odorranalectin

**Figure 6:**
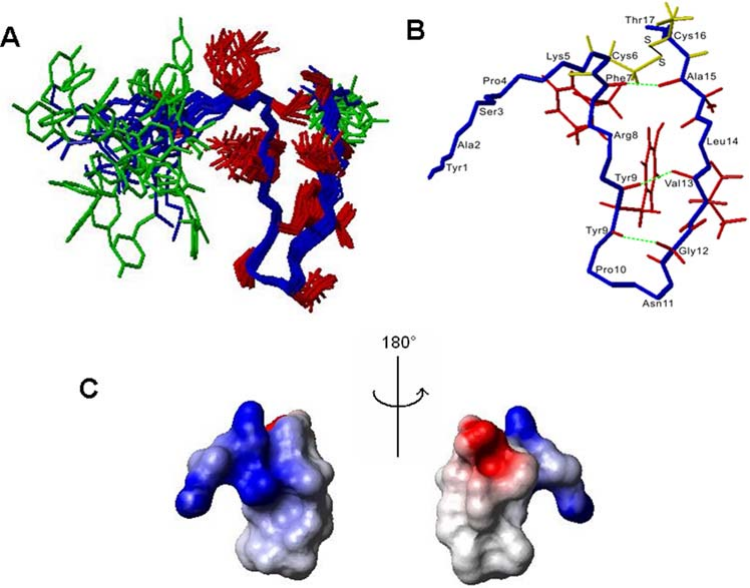
The solution structure of odorranalectin. (A) Superposition of the 20 lowest-energy conformers calculated with NOE-derived ^1^H-^1^H distance restraints and J-coupling restraints. Backbone, side-chains of residues 4–16 and side-chains of residues 1–3, 4 were shown in blue, red and green, respectively. (B) The mean structure calculated from the 20 lowest-energy structures which highlighted three hydrogen bonds (green broken lines) and one disulfide bonds (black solid line). (C) Electrostatic surface of odorranalectin which took the same orientation as that in the Panel A. Positively charged region and negatively charged region were shown in blue and red, respectively. The electrostatic surface was calculated and colored using the program MOLMOL [31].

Proton nuclear magnetic resonance (NMR) spectroscopy was used to determine the three-dimensional structure of odorranalectin in the NMR buffer described in the section of Materials and Methods (Fig. 6, SMSDep ID code: 20001; BMRB ID code: 15556). ^1^H resonance assignments were performed using TOCSY and NOESY spectra for identification of the scalar coupled spin systems and the sequential connectivity. Totally, 187 ^1^H-^1^H distance constraints were obtained, including 101 intraresidue constraints, 45 sequential constraints and 29 medium constraints, 12 long range constraints. NOE distance constraints were shown in Tables S8, while J-coupling constraints were shown in Tables S9 which was obtained from TOCSY spectrum. ^1^H chemical shifts of odorranalectin were listed in Table S10. The structure statistics for the 20 lowest-energy structures of 200 calculated structures was shown in Table S11. The average RMSD (root-mean-square deviation) values for the backbone atoms and for all heavy atoms of residues 4–16 are 0.55 Å and 0.93 Å, respectively, calculated by the program MOLMOL [16]. The Xxx-Pro peptide bonds with Pro4 and with Pro10 both are in the *trans*-conformation, as evidenced by the observation of d_αδ_ NOEs. Ramachandran plot analysis using the program PROCHECK [17] showed that 60% residues were located in the most favored region, while 35.4% residues were located in the additional allowed regions. No residue was located in the disallowed region.

The cyclic peptide of odorranalectin adopts a β-turn conformation which is stabilized by one intramolecular disulfide bond between Cys6-Cys16 and three hydrogen bonds between Phe7-Ala15, Tyr9-Val13, Tyr9-Gly12 (Fig.6B). It was revealed that there were no secondary structural elements such as helix, strand in the NMR-derived conformation, which was consistent with the result from the circular dichroism (CD) experiment (Fig. S2). Furthermore, both termini (residues Tyr1-Ser3, Tyr17) are quite conformational flexible and were not included in the RMSD calculation. Most regions of the electrostatic surface of odorranalectin (Fig. 6C) are positively charged, except the region around T17 (Fig. 6C) is negatively charged. The solution structure disclosed by the NMR study provides a molecular basis for understanding the function of odorranalectin.

## Discussion

Together, odorranalectin identified from skin secretions of the frog of *O. grahami* represents a new family of lectin. Odorranalectin can thus be added to the growing list of amphibian skin bioactive peptide prototypes. Amphibian skin secretions are a rich source of biologically active peptides which participate in mechanisms used by frogs and toads to defend against microbial infection and from being eaten by predators [18], [19]. Antimicrobial peptides and lectins generally belong to families of bioactive peptides, which through evolution have given rise to counterparts in microorganisms. Odorranalectin is the smallest lectin, which is composed of only 17 amino acid residues. The overall structure of odorranalectin precursor is very similar to amphibian antimicrobial peptide precursors [9], and odorranalectin could synergize with amphibian antimicrobial peptides. All the results imply that amphibian antimicrobial peptides and odorranalectin have the same ancestor and odorranalectin takes part in antimicrobial defence as antimicrobial peptides do. It also disclosed a new strategy of amphibian anti-infection.

Although many scientific problems and technical developments need to be solved, lectin-mediated oral drug delivery is more than an interesting idea. Most lectins are large and are unlikely to be used as drug carriers because of their immunogenicity and toxicity [1]. Smaller peptides or even organic molecules which can mimic the function of lectins are ideal candidates [1]. As the smallest lectin, odorranalectin has potential for drug delivery and targeting because of its several unique characters. Firstly, odorranalectin is composed of only 17 amino acid residues with a molecular weight of 1887.9 Da and has a single disulfide bridge that makes it easy to manipulate and develop a drug targeting system. Secondly, odorranalectin can specifically bind to monomeric sugar, L-fucose, that makes odorranalectin have the ability to target special sites, and odorranalectin was proved to mainly conjugate to liver, spleen and lung after iv administration. Thirdly, it was apparently stable for more than 5 hours in mice plasma that can improve its bioavailability. Lastly, it is very important that odorranalectin has extremely low toxicity and immunogenicity.

## Materials and Methods

### Materials and animals

All the information for materials and animals are provided in supplementary materials. The animals used for the experiment were treated according to the protocols evaluated and approved by the ethical committee of Kunming Institute of Zoology.

### Lectin purification, construction and screening of a cDNA library

Lectin purification, mRNA preparation, cDNA library construction and screening were performed as described previously [9]. The details are provided in the supplementary materials (Materials and Methods S1).

### Peptide synthesis

Odorranalectin and its mutants synthesis and ^125^I-odorranalectin preparation with iodogen methods were performed as described previously [9], [20]. The details are provided in the supplementary materials (Materials and Methods S1; Figure S3).

### Erythrocytes hemagglutination and inhibition assay

Intact, pronase-treated, trypsin-treated, and formaldehyde-treated rabbit erythrocytes were prepared. Assays were carried out in U-well microtiter plates (96 wells) as described previously [21]–[23]. The details are provided in the supplementary materials (Materials and Methods S1).

### Microbe-agglutinating activities

The bacteria agglutination assay was performed according the method described by Yu et al [24]. The details are provided in the supplementary materials (Materials and Methods S1).

### SPR Analysis

Real time detection of odorranalectin and its mutants binding to glycoproteins and L-fucose was recorded by using a Biacore 3000 [21]–[24]. The details are provided in the supplementary materials (Materials and Methods S1).

### Histamine release activities

Histamine release activity was assayed according to the methods described by Shore et al [25]. The details are provided in the supplementary materials (Materials and Methods S1).

### Stability of ^125^I-odorranalectin *in vitro*

In order to evaluate the stability of ^125^I- odorranalectin *in vitro*, a degradation experiment of ^125^I-odorranalectin in mice plasma was performed. The details are provided in the supplementary materials (Materials and Methods S1).

### Tissue distribution of ^125^I-odorranalectin *in vivo*

Sixty-three female KM mice (18∼22 g) were grouped randomly into three groups receiving oral, intravenous and intranasal administration of ^125^I-odorranalectin at equal radiation dose (400 µCi·kg^−1^), respectively. The detail procedures are provided in the supplementary materials (Materials and Methods S1).

### Immunogenicity and toxicity assay

Immunogenicity and toxicity were tested according to methods described by Scott *et al* [26]. Groups of mice (n = 10, seven-week-old, female) were immunized by odorranalectin and other control proteins (ovalbumin and wheat germ agglutinin), using 0.1 M sodium bicarbonate as blank control. After three immunizations, the detection of specific antibodies in sera was performed by enzyme-linked immunosorbent assay (ELISA).

### Nuclear magnetic resonance (NMR)

The NMR sample was prepared by dissolving 2.9 mg odorranalectin in 500 µl NMR buffer including 20 mM Na_2_HPO_4_, 100 mM NaCl, pH 6.0. All NMR experiments and circular dichroic analysis were performed according to previous methods [16], [17], [27]–[32]. The details were described in the supplementary materials (Materials and Methods S1).

### NMR titration Experiments

Titration of L-fucose to odorranalectin was performed as follows. A sample of 10 mM odorranalectin in the HBS-EP buffer (10 mM HEPES, 150 mM NaCl, 3 mM EDTA, 0.005% surfactant P-20, pH 7.4, in 90% H_2_O/10% D_2_O), was titrated with 90 mM L-fucose in the HBS-EP buffer too. Data were collected with 64 and 1024 complex data points in t1 and t2 dimensions, respectively, and signals were averaged over 128 transients. At each titration point, 2D ^1^H-^15^N HSQC spectrum was recorded for bound odorranalectin at molar ratios of odorranalectin to L-fucose of 1∶0.25, 1∶0.5, 1∶0.75, 1∶2, 1∶3. Resonance assignments were achieved using a combination of TOCSY, NOESY and ^1^H-^15^N HSQC spectra.

### Data analysis

Each experiment was carried out in triplicate. Values are given as means±SD. All the concentration data were plotted directly as radiocounting-time curves of the blood and tissues, and the area under the curve (AUC_0→t_) was calculated by the trapezoidal rule. The statistical differences between per oral or intranasal administrations and intravenous administration were assessed using student's *t* test and *P*<0.05 as statistical significance.

## Supporting Information

Materials and Methods S1 Detailed materials and methods
(0.06 MB DOC)

Figure S1 Purification of odorranalection from O. grahami skin secretion by a Sephadex G-50 gel filtration (A) and Hypersil BDS C18 RP-HPLC (B, C).
(0.19 MB TIF)

Figure S2 A: The CD spectrum of odorranalectin in NMR buffer (20 mM Na2HPO4, 100 mM NaCl, pH6.0). B: The estimated populations of secondary structure elements contained in odorranalectin.
(0.05 MB DOC)

Figure S3 HPLC of 125I-Lectin X. A: UV detector; B: radioactive detector (before purification); C: radioactive detector (after purification)
(0.18 MB DOC)

Table S1 Erythrocytes agglutination profiles of odorranalectin.
(0.03 MB DOC)

Table S2 Microorganism agglutination profiles of odorranalectin
(0.03 MB DOC)

Table S3 Histamine releasing activity of odorranalectin
(0.02 MB DOC)

Table S4 Inhibition and binding kinetics of odorranalectin-mediated erythrocytes hemagglutination by mono- and oligosaccharides and glycoproteins
(0.03 MB DOC)

Table S5 Abilities of odorranalectin and its mutants to bind with L-fucose
(0.03 MB DOC)

Table S6 Area under radiocounting-time curves (AUC) of blood and tissues after per oral, transvenous and per nasal administrations into mice at 400 µCi.kg-1 dose (Mean±SD, n = 3)
(0.03 MB DOC)

Table S7 Radiochemical purity (%) of 125I-odorranalectin determined before and after purification (n = 3)
(0.03 MB DOC)

Table S8 NOE restraints (upper limits of 1H-1H distances, Å) used for structure calculation of odorranalectin
(0.32 MB DOC)

Table S9 3J (HN-HA) scalar coupling constants (Hz) used for structure calculation of odorranalectin as backbone dihedral angle restraints
(0.03 MB DOC)

Table S10 ^1^H chemical shifts of odorranalectin in H_2_O at 298 K
(0.03 MB DOC)

Table S11 Structure statistics from NMR structure analysis
(0.04 MB DOC)
