# Supplementary material for: Odorranalectin Is a Small Peptide Lectin with Potential for Drug Delivery and Targeting
Source: PLoS One. 2008 Jun 11;3(6):e2381. doi: 10.1371/journal.pone.0002381 (PMC2440032; doi:10.1371/journal.pone.0002381)
Supplement: Materials and Methods S1 — Detailed materials and methods (0.06 MB DOC) [file pone.0002381.s001.doc]

**Supplementary materials**

**METHODS**

**Materials and animals.** Adult specimens of *Odorrana grahami* were collected in Kunming, Yunnan Province, South West of PR China. *N*-glycolylneuraminic acid (NeuGc), *N*-acetylneuraminic acid (NeuAc) and *N-*acetylmannosamine were purchased from Guangzhou Junkang Co Ltd. Bovine submaxillary mucin (BSM), porcine stomach mucin (PSM), and Fetuin were purchased from Sigma. All other sugars and glycoproteins for the sugar analysis, the hemaaggluting inhibition tests and the SPR analysis were products of Solarbora or Calbiochem. The BIAcore 3000 was a product of BIAcore AB (Sweden), deoxyribonucleotides for cyclic thermal amplification were purchased from Takara (Japan).

KM mice (18-22 g, ♀) were obtained from Experimental Animal Center of Kunming and maintained under standard conditions with access to food and water ad libitum. The animals used for the experiment were treated according to the protocols evaluated and approved by the ethical committee of Kunming Institute of Zoology, Chinese Academy of Sciences.

**Construction and screening of a cDNA library.** mRNA preparation, cDNA library construction and screening were performed as described before [7]. Skin secretions collected from *O. grahami* were fractionated by Sephadex G-50 (Superfine, Amersham Biosciences, 2.6 × 100 cm). The fraction VI (Fig. S1A) containing lectin-like activity was purified further by C_18_ reverse phase high performance liquid chromatography (RP-HPLC, Hypersil BDS C_18_, 30 × 0.46 cm) column as illustrated in Fig. S1B and Fig. S1C. The purified lectin (odorranalectin) was indicated by an arrow. Complete peptide sequencing was undertaken by Edman degradation on an Applied Biosystems pulsed liquid-phase sequencer, model 491.

Total RNA was extracted using TRIzol (Life Technologies, Ltd.) from the skin of single sample of *O. grahami.* cDNA was synthesized by SMART^TM^ techniques by using a SMART^TM^ PCR cDNA synthesis kit (Clontech, Palo Alto, CA). The cDNA synthesized by SMART^TM^ techniques was used as a template for PCR to screen the cDNAs encoding odorranalectin. Two oligonucleotide primers, S_1_ 5’- TA(T/C)GC(A/T/C/G)AG(T/C)CG(A/T/C/G)AA(A/G)TG(T/C)TT(T/C)AG(A/G)-3’, in the reverse direction, a specific primer designed according to the amino acid sequence determined by Edman degradation and primer II A provided by the SMART^TM^ PCR cDNA synthesis kit in the sense direction were used in PCR reactions. The DNA polymerase was Advantage polymerase from Clontech (Palo Alto, CA). The PCR conditions were: 2 min at 94^o^C, followed by 30 cycles of 10 sec at 92^o^C, 30 sec at 50^o^C, 40 sec at 72^o^C. Finally, the PCR products were cloned into pGEM®-T Easy vector (Promega, Madison, WI). DNA sequencing was performed on an Applied Biosystems DNA sequencer, model ABI PRISM 377.

**Peptide synthesis.** Odorranalectin and its mutants were synthesized by AC SCIENTIFIC (Xi An) INC (Xi An, China) and analyzed by HPLC and MALDI-TOF mass spectrometry to confirmed purity higher than 95%. ^125^I-odorranalectin was prepared with iodogen methods as described previously [20]. The labeled product was purified by an anion-exchange resin microcolumn centrifuge method to remove ^125^I^-^ free ion. Radiolabeling efficiency and radiochemical purity of ^125^I-odorranalectin eluate were determined with silica gel paper chromatography (SGPC) and high-performance liquid chromatography (HPLC).

**Erythrocytes hemagglutination and Inhibition Assay.** Intact, pronase-treated, trypsin-treated, and formaldehyde-treated rabbit erythrocytes were prepared as described previously [21-23]. Assays were carried out in U-well microtiter plates (96 wells). The hemagglutinating activity of the lectin was determined by a 2-fold serial dilution procedure using intact, pronase-treated, trypsin-treated, formaldehyde-treated rabbit erythrocytes and three strains of bacteria, the hemagglutination titer was defined as the reciprocal of the highest dilution exhibiting hemagglutination.

To examine divalent cation requirements, 20 mM Tris–HCl (pH 7.5) and 150 mM NaCl (TBS) containing 50 mM EDTA or 50 mM CaCl_2_ was used as the assay buffer. To determine specific saccharides or glycoprotein, inhibition of hemagglutinating activity via the addition of saccharides was tested by adding 2-fold serial dilutions of test saccharide or glycoprotein to wells containing the synthesized odorranalectin using TBS as the assay buffer. Inhibition was expressed as the minimum concentration of each sugar or glycoprotein required for inhibition of hemagglutination of titer 4 of the lectin.

**Bacteria agglutinating activities.** The bacteria agglutination assay was performed according the method described by Yu et al [24]. *Escherichia coli* DH5a*, Staphylococcus aureus* (ATCC2592) and *Candida albicans* (ATCC 2002) were cultured overnight in nutrient broth. After incubation, these bacteria were centrifuged at 3000 rpm for 5 min and washed twice with PBS, and then were resuspended in TBS at a cell density of 5.6 ×10^9^ organisms /ml.

Odorranalectin was two-fold serially diluted with TBS. In each 500 μl microfuge tube, 25 μl of the tested bacteria suspension was mixed with 25 μl of the diluted lectin. The tubes were left at room temperature for 45 min and were then shaken at full speed on mixer for 20 s. One drop of the mixture was then placed on a slide and covered with a coverslip. The agglutination was examined under a light microscope with dark-field illumination.

**SPR Analysis.** Real time detection of odorranalectin and its mutants binding to glycoproteins and L-fucose was recorded by using a Biacore 3000 [21-24]. Odorranalectin was immobilized covalently via their primary amines to carboxyl groups within a dextran layer on the sensor chip CM-5 according to the manufacturer’s specifications. After chip activation with 0.4 M 1-ethyl-3- (3-dimethylaminopropyl) carbodiimide hydrochloride and 10 mM *N*-hydroxysuccinimide, odorranalectin or its mutants (in 10 mM sodium acetate buffer, pH 4.0) at a concentration of 100 μg/ml was passed through the flow cells at a rate of 5 μl/min. After immobilization, the chip was capped by exposure to 1 M ethanolamine (pH 8.5).

All analyses were performed at a flow rate of 30 μl/min. Before loading of analytes, the chip was equilibrated with 10 mM HEPES-buffered saline containing 3 mM EDTA and 0.005% surfactant P-20, pH 7.4 (HBS-EP). Each analyte at various concentrations in the same buffer was injected over the immobilized ligand for 4 min. After injection of the analyte, HBS-EP was introduced onto the sensor surface to start dissociation, and dissociation data were collected for 5 min. The chip was regenerated by injection of 5μl of 10 mM HCl or NaOH, followed by HBS-EP.

Association and dissociation rate constants (*ka* and *kd*) were calculated by using BIA evaluation 4.0 software (Biacore AB, Sweden). The affinity constant (*KD*) was calculated from the *ka* and *kd*. For the calculation of rate constants, samples were appropriately diluted in HBS-EP at various concentrations.

**Histamine release activities.** Male or Wistars rats (150–250 g body weight) were lightly anaesthetized with CO_2_ and then killed by cervical dislocation and exsanguination. Mixed peritoneal cells were obtained and the cells were washed twice in Tyrode's buffer (NaCl (137 nM) glucose (5.6 mM), HEPES (10 mM), KCl (2.7 mM), MgCl_2_·6H_2_O (1 mM), CaCl_2_·2H_2_O (1 mM) and NaH_2_PO_4_·2H_2_O (0.4 mM), pH 7.4) and recovered by centrifugation (100 g, 4 °C, 2 min). Isolated peritoneal cells (100 μl) were aliquoted into conical polystyrene test tubes and prewarmed to 37 °C for 5 min. Synthesized odorranalectin was reconstituted in Tyrode's buffer (100 μl) and added to cells suspensions. Following incubation (10 min, 37 °C), the reaction was quenched by addition of ice-cold Tyrode's buffer (2.8 ml). The cell suspensions were centrifuged as above and the supernatants removed for histamine assay. The remaining pellets were resuspended in buffer (3 ml) and then placed in a boiling water bath (10 min) to release the residual histamine. The histamine content was determined in both the supernatants and the cell pellets using the fluorimetric method based on Shore et al [25]. Histamine release was expressed as the percentage of total content and values were corrected for spontaneous release in the absence of peptides not exceeding 5%.

**Circular dichroism(CD) spectroscopy.**

The far-UV CD experiments were performed on a Jasco J-810 spectrometer (Jasco, Japan) to investigate the secondary structure of odorranalectin, using a cylindrical cell of 0.1 cm path-length, 2-nm bandwidth, 1-s response time, and a scan speed of 200 nm/min. Samples were prepared by dissolving the peptide to the concentration of 70 μM in NMR buffer (20 mM Na_2_HPO_4_, 100 mM NaCl, pH 6.0). The spectra were measured between 190 and 260 nm, three consecutive scans per sample were performed at 25 ^o^C. Three scans were added and averaged, followed by subtraction of the CD signal of the NMR buffer. The percentages of secondary structure elements were estimated according to the J.T. Yang formula [27].

**Stability of ^125^I-odorranalectin *in vitro*.** In order to evaluate the stability of ^125^I- odorranalectin *in vitro*, a degradation experiment of ^125^I-odorranalectin in mice plasma was performed. Briefly, 5 µCi ^125^I-odorranalectin (about 10 µl) was added to 790 µl plasma (100%, v/v) freshly prepared and 400 µL plasma (50%) admixed with 390 µl PBS (pH 7.4) in two 1.5-ml centrifuge tubes, respectively, which maintained at 37 °C in a rolling water-bath incubator. Then at each time point (0, 1, 2, 3, 4 and 5 h), a glass microcap was used to take samples for silica gel paper chromatography (SGPC) analysis and high-performance liquid chromatography (HPLC) for calculating the radiochemical purity of ^125^I-odorranalectin. In detail, the developing agent of SGPC was methanol: water = 85:15 (v/v); After spotted and the solvent reached the frontier, the silica gel paper strip was dried and cut to ten equations (Fig. S3 and Table S7), and then set in 10 tubes, following determined radiocounting of each piece in a SN-695 Intellectual Radio-immunity γ Measurement (Shanghai Institute of Nuclear Research Ri Huan Instrument Factory, China), respectively. Rf values of ^125^I-odorranalectin and free ^125^I^-^ were 0-0.1 and 0.7-0.9, respectively. The HPLC method consisted of a UV detector at 220 nm (SPD-10A Vp, Shimadzu, Japan) and a radioactive detector SN-682 γ Counter (Shanghai Institute of Nuclear Research Ri Huan Instrument Factory, China) in series used in liquid chromatography (LC-10 AT Vp, Shimadzu, Japan) and a workstation (EChrom 98, Dalian Elite, China). A Jupiter^TM^ 300 C4 Phenomenex® column (250 ×4.6 mm, 5 μ, USA) was used for the separation and eluted with a linear gradient 0-100% (v/v) acetonitrile (0.1% trifluoroacetic acid) in 20 min with 1.0 ml/min flow rate at room temperature.

**Tissue distribution of ^125^I-odorranalectin *in vivo*.** Sixty-three female KM mice (18~22 g) were grouped randomly to three groups receiving oral, intravenous and intranasal administration at equal radiation dose (400 µCi·kg^-1^), respectively. The procedure for intranasal administration was performed gently and slowly so as to allow the mice to inhale all of the preparations (each nostril 10 µl). At different time intervals (5, 15, 30, 60, 120, 180 and 300 min) following administration, blood sample was collected via eye puncture; subsequently, the mice were humanly sacrificed, and tissues such as heart, liver, spleen, lung, stomach (content removed), kidney and brain were harvested and stuffed into a special tube after the remnant blood was blotted by tissue paper, following weighing, respectively. Finally, the radiocounting of each tissue was determined in a SN-695 Intellectual Radio-immunity γ Measurement to evaluate odorranalectin intake.

**Nuclear magnetic resonance (NMR).** The NMR sample was prepared by dissolving 2.9 mg odorranalectin in 500 μl NMR buffer including 20 mM Na_2_HPO_4_, 100 mM NaCl, pH 6.0. All NMR experiments were performed on a Varian Unity INOVA 600 MHz spectrometer equipped with three RF channels and a triple resonance z-axis pulsed-field gradient probe. Two-dimensional phase-sensitive NMR spectra, including TOCSY (mixing time of 75 ms) and NOESY (mixing time of 300 ms), were recorded at 298 K. The watergate approach was employed for suppression of the solvent peak. Quadrature detection in the F1 dimension was achieved using the states-TPPI approach. Data were collected with 256 and 1024 complex data points in t1 and t2 dimensions, respectively, and signals were averaged over 32 transients. Data were processed using the program NMRPipe/NMRDraw and analyzed by Sparky [16, 17]. Linear prediction in the t1 dimension was used before the Fourier transformation. Proton chemical shifts were referenced to DSS [30]. Structure calculations were performed according to the standard ARIA protocol [31] by using CNS 1.1 software [3[2](http://www.pnas.org/cgi/content/full/99/22/14053?maxtoshow=&HITS=10&hits=10&RESULTFORMAT=&fulltext=nmr+aria&searchid=1&FIRSTINDEX=0&resourcetype=HWCIT#B28%23B28)]. A family of 200 structures were calculated and 20 lowest-energy structures were selected, which exhibited no NOE violation ＞0.3 Å. Ramachandran plot analysis was performed using the program PROCHECK [16].

**Data analysis.** Each experiment was carried out in triplicate. Values are given as means ± SD. All the concentration data were plotted directly as radiocounting-time curves of the blood and tissues, and the area under curve (AUC_0→t_) was calculated by the trapezoidal rule. The statistical differences between per oral or intranasal administrations and intravenous administration were assessed using student’s *t* test and *P*< 0.05 as statistical significance.
