# Supplementary figures and images for: Odorranalectin Is a Small Peptide Lectin with Potential for Drug Delivery and Targeting
Source: PLoS One. 2008 Jun 11;3(6):e2381. doi: 10.1371/journal.pone.0002381 (PMC2440032; doi:10.1371/journal.pone.0002381)

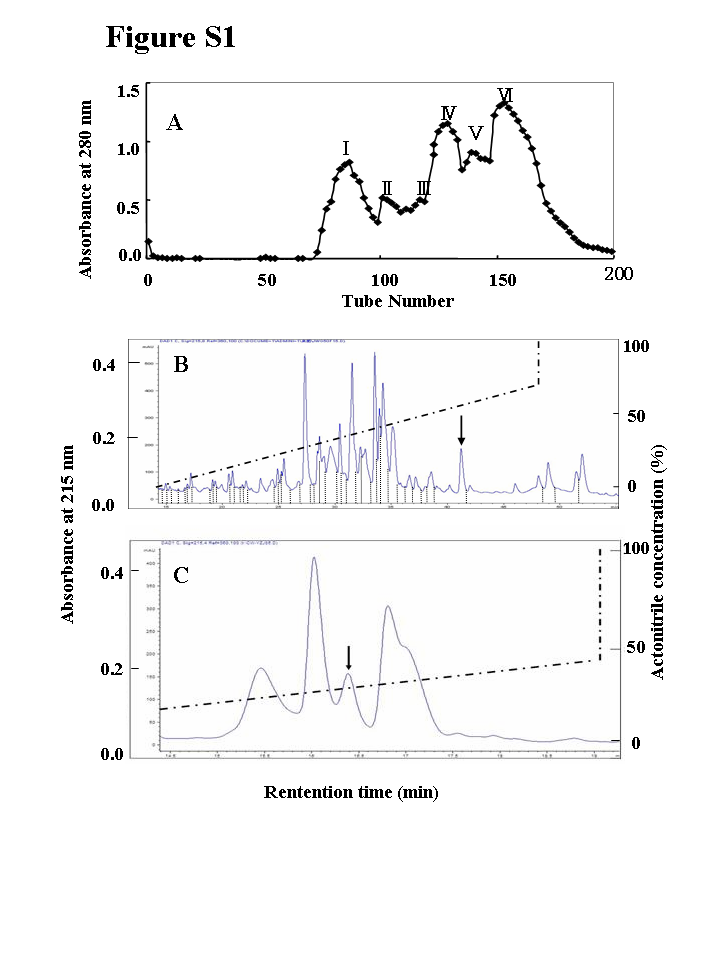

Supplement: Figure S1 — Purification of odorranalection from O. grahami skin secretion by a Sephadex G-50 gel filtration (A) and Hypersil BDS C18 RP-HPLC (B, C). (0.19 MB TIF) [file pone.0002381.s002.tif]

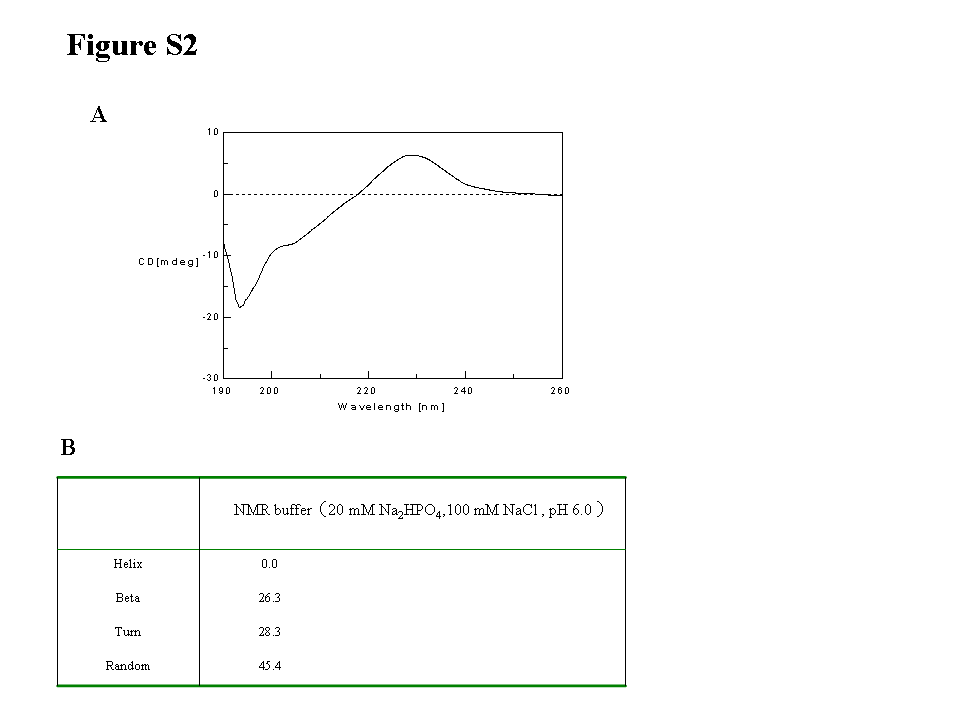

Supplement: Figure S2 — A: The CD spectrum of odorranalectin in NMR buffer (20 mM Na2HPO4, 100 mM NaCl, pH6.0). B: The estimated populations of secondary structure elements contained in odorranalectin. (0.05 MB DOC) [file pone.0002381.s003.tif]

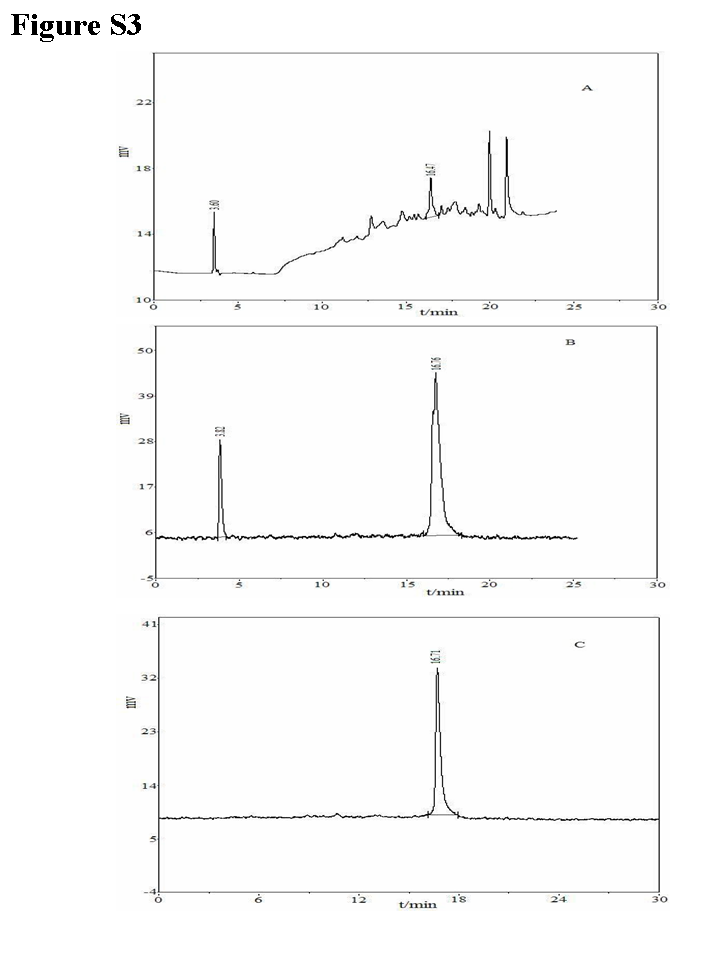

Supplement: Figure S3 — HPLC of 125I-Lectin X. A: UV detector; B: radioactive detector (before purification); C: radioactive detector (after purification) (0.18 MB DOC) [file pone.0002381.s004.tif]
