# Supplementary material for: Odorranalectin Is a Small Peptide Lectin with Potential for Drug Delivery and Targeting
Source: PLoS One. 2008 Jun 11;3(6):e2381. doi: 10.1371/journal.pone.0002381 (PMC2440032; doi:10.1371/journal.pone.0002381)
Supplement: Table S1 — Erythrocytes agglutination profiles of odorranalectin. (0.03 MB DOC) [file pone.0002381.s005.doc]

Table S1 Erythrocytes agglutination profiles of odorranalectin.

Concentration (μg/ml)

Erythrocyte Untreated pronase-treated^a^ trypsin-treated^b^ formaldehyde-treated

Human A 0.75

Human B 0.75

Human O 1.12

Human AB 1.12

Rat 1.12

Sheep 1.12

Quail 3

Rabbit 1.12 5.25 1.12 1.12

*^a^* 10% suspension of erythrocytes in PBS (10 ml) was treated with Pronase (5.0 mg) for 30 min at 45 °C;

*^b^* 10% suspension of erythrocytes in PBS (10 ml) was treated with trypsin (1.0 mg) for 180 min at 37 °C.
