# Supplementary material for: Odorranalectin Is a Small Peptide Lectin with Potential for Drug Delivery and Targeting
Source: PLoS One. 2008 Jun 11;3(6):e2381. doi: 10.1371/journal.pone.0002381 (PMC2440032; doi:10.1371/journal.pone.0002381)
Supplement: Table S2 — Microorganism agglutination profiles of odorranalectin (0.03 MB DOC) [file pone.0002381.s006.doc]

Table S2 Microorganism agglutination profiles of odorranalectin

Bacteria MIC (μg/ml)

*E. Coli DH5a,* 0.625

*S. aureus* (*ATCC2592*) 2.24

*C. albicans* (*ATCC2002*) 1.12

MIC is the minimum concentration to induce microorganism agglutination
