# Supplementary material for: Odorranalectin Is a Small Peptide Lectin with Potential for Drug Delivery and Targeting
Source: PLoS One. 2008 Jun 11;3(6):e2381. doi: 10.1371/journal.pone.0002381 (PMC2440032; doi:10.1371/journal.pone.0002381)
Supplement: Table S3 — Histamine releasing activity of odorranalectin (0.02 MB DOC) [file pone.0002381.s007.doc]

Table S3 Histamine releasing activity of odorranalectin

Concentration（μg/ml） histamine release rate(%)

0 0

25 5.72±1.7

50 12.48±4.9
