# Supplementary material for: Odorranalectin Is a Small Peptide Lectin with Potential for Drug Delivery and Targeting
Source: PLoS One. 2008 Jun 11;3(6):e2381. doi: 10.1371/journal.pone.0002381 (PMC2440032; doi:10.1371/journal.pone.0002381)
Supplement: Table S4 — Inhibition and binding kinetics of odorranalectin-mediated erythrocytes hemagglutination by mono- and oligosaccharides and glycoproteins (0.03 MB DOC) [file pone.0002381.s008.doc]

Table S4 Inhibition and binding kinetics of odorranalectin-mediated erythrocytes hemagglutination by mono- and oligosaccharides and glycoproteins.

___________________________________________________________________

Analytes MIC *ka kd K_D_*

*M*^-^*^1^ s*^-^*^1^ s*^-^*^1^ M*

BSM 32.5 μg/ml 332 9.65×10^-4^ 2.9×10^-6^

Fetuin 15.6 μg/ml 2.89×10^3^ 6.1×10^-4^ 2.11×10^-7^

PSM 93.7 μg/ml 723 1.38×10^-3^ 1.91×10^-6^

L-fucose 12.5 mM 159 8.67×10^-3^  5.47×10^-5^

MIC is the minimum concentration to inhibit odorranalectin-mediated erythrocytes hemagglutination.
