# Supplementary material for: Odorranalectin Is a Small Peptide Lectin with Potential for Drug Delivery and Targeting
Source: PLoS One. 2008 Jun 11;3(6):e2381. doi: 10.1371/journal.pone.0002381 (PMC2440032; doi:10.1371/journal.pone.0002381)
Supplement: Table S5 — Abilities of odorranalectin and its mutants to bind with L-fucose (0.03 MB DOC) [file pone.0002381.s009.doc]

Table S5 Abilities of odorranalectin and its mutants to bind with L-fucose

________________________________________________________________

Samples  Sequences  binding affinity (*K*_D_)

Odorranalectin YASPKCFRYPNGVLACT 5.47×10^-5^ M

Odorranalectin K5/G5 YASPGCFRYPNGVLACT NA

Odorranalectin C6/G6 YASPGGFRYPNGVLACT NA

Odorranalectin F7/G7 YASPGGGRYPNGVLACT NA

Odorranalectin T17/G17 YASPGGFRYPNGVLACT 8.2×10^-3^ M

NA: No binding to L-fucose
