# Supplementary material for: Odorranalectin Is a Small Peptide Lectin with Potential for Drug Delivery and Targeting
Source: PLoS One. 2008 Jun 11;3(6):e2381. doi: 10.1371/journal.pone.0002381 (PMC2440032; doi:10.1371/journal.pone.0002381)
Supplement: Table S6 — Area under radiocounting-time curves (AUC) of blood and tissues after per oral, transvenous and per nasal administrations into mice at 400 µCi.kg-1 dose (Mean±SD, n = 3) (0.03 MB DOC) [file pone.0002381.s010.doc]

Table S6 Area under radiocounting-time curves (AUC) of blood and tissues after per oral, transvenous and per nasal administrations into mice at 400 µCi·kg^-1^ dose (Mean ± SD, n=3)

___________________________________________________________________________________________________________

AUC / (Mean ± SD)*10^7^ CPM·min

___________________________________________________________________________________________________________

Routes Blood Brain Heart Liver Spleen Lung Kideny Stomach

po ^a^ 4.89±0.69 0.25±0.024* 1.99±0.24 2.42±0.29** 3.60±0.35** 4.02±0.51** 3.84±0.76* 126.64±32.27**

iv 5.41±1.11 0.46±0.047 3.37±0.51 98.51±6.22 64.82±8.61 246.41±62.56 8.47±1.41 20.90±3.09

in ^b^ 6.77±3.05 0.36±0.15 2.88±1.16 3.53±1.50** 4.48±1.86** 12.50±3.77** 5.17±2.10 125.61±44.28**

^a^:* p<0.05; ** P<0.01, po *vs* iv. ^b^: * p<0.05; ** P<0.01, in *vs* iv.
