# Supplementary material for: Odorranalectin Is a Small Peptide Lectin with Potential for Drug Delivery and Targeting
Source: PLoS One. 2008 Jun 11;3(6):e2381. doi: 10.1371/journal.pone.0002381 (PMC2440032; doi:10.1371/journal.pone.0002381)
Supplement: Table S7 — Radiochemical purity (%) of 125I-odorranalectin determined before and after purification (n = 3) (0.03 MB DOC) [file pone.0002381.s011.doc]

Table S7. Radiochemical purity (%) of ^125^I-odorranalectin determined before and after purification (n=3)

| No. | Radiocounting (Counts Per Minute, CPM) | |
| --- | --- | --- |
|  | Before purification | After purification |
| 1 | 290424/135419/217398 | 137964/103188/153180 |
| 2 | 35748/7936/18648 | 21846/9462/13446 |
| 3 | 4200/2025/10194 | 11748/4110/7410 |
| 4 | 5688/1731/5922 | 4974/1998/4896 |
| 5 | 5952/2502/3192 | 3036/1062/2490 |
| 6 | 7188/3107/2256 | 2298/1008/2124 |
| 7 | 6282/3557/2574 | 2298/1008/1818 |
| 8 | 17250/10137/5454 | 2790/2448/2556 |
| 9 | 67644/33117/26304 | 5208/5832/8430 |
| 10 | 20106/8437/10632 | 3030/3108/5100 |
| Radiochemical purity (%) ^a^ | 78.0 ± 6.53 | 92.4 ± 0.702 |

^a^: Radiochemical purity (%) = CPM_1_/(CPM_1_+ CPM_7-9_)*100
