# Supplementary material for: Odorranalectin Is a Small Peptide Lectin with Potential for Drug Delivery and Targeting
Source: PLoS One. 2008 Jun 11;3(6):e2381. doi: 10.1371/journal.pone.0002381 (PMC2440032; doi:10.1371/journal.pone.0002381)
Supplement: Table S8 — NOE restraints (upper limits of 1H-1H distances, Å) used for structure calculation of odorranalectin (0.32 MB DOC) [file pone.0002381.s012.doc]

Table S8 NOE restraints (upper limits of ^1^H-^1^H distances, Å) used for structure calculation of odorranalectin.

| Residue number | Residue name | Atom name | Residue number | Residue name | Atom name | Distance |
| --- | --- | --- | --- | --- | --- | --- |
| 1 | TYR | QD | 1 | TYR | QE | 3.76 |
| 3 | SER | HB2 | 3 | SER | HA | 2.90 |
| 3 | SER | HN | 3 | SER | HB2 | 4.68 |
| 4 | PRO | HB2 | 4 | PRO | HA | 4.25 |
| 4 | PRO | HB2 | 4 | PRO | HD2 | 3.38 |
| 4 | PRO | HB3 | 4 | PRO | HA | 3.27 |
| 4 | PRO | HD2 | 3 | SER | HA | 3.38 |
| 4 | PRO | HD2 | 4 | PRO | HG2 | 3.13 |
| 4 | PRO | HD2 | 4 | PRO | HG3 | 3.86 |
| 4 | PRO | HG3 | 4 | PRO | HG2 | 3.12 |
| 4 | PRO | HG3 | 4 | PRO | HG3 | 2.49 |
| 5 | LYS+ | HB2 | 5 | LYS+ | HA | 3.01 |
| 5 | LYS+ | HB2 | 5 | LYS+ | HN | 3.70 |
| 5 | LYS+ | HB3 | 5 | LYS+ | HA | 3.14 |
| 5 | LYS+ | HB3 | 5 | LYS+ | HB2 | 2.17 |
| 5 | LYS+ | HB3 | 5 | LYS+ | HN | 3.75 |
| 5 | LYS+ | QE | 6 | CYS | HN | 2.93 |
| 5 | LYS+ | HG2 | 5 | LYS+ | HB3 | 4.36 |
| 5 | LYS+ | HG2 | 5 | LYS+ | QD | 2.69 |
| 5 | LYS+ | HG3 | 5 | LYS+ | HB3 | 3.60 |
| 5 | LYS+ | HN | 4 | PRO | HA | 3.03 |
| 5 | LYS+ | HN | 4 | PRO | HB3 | 4.68 |
| 5 | LYS+ | HN | 5 | LYS+ | HA | 3.71 |
| 5 | LYS+ | HN | 5 | LYS+ | HG3 | 4.70 |
| 5 | LYS+ | HN | 6 | CYS | HN | 4.27 |
| 6 | CYS | HA | 6 | CYS | HB2 | 3.17 |
| 6 | CYS | HA | 7 | PHE | HN | 2.73 |
| 6 | CYS | HB3 | 8 | ARG+ | HG3 | 3.56 |
| 6 | CYS | HB3 | 14 | LEU | QD2 | 4.21 |
| 6 | CYS | HN | 5 | LYS+ | HA | 2.87 |
| 6 | CYS | HN | 5 | LYS+ | HB2 | 4.41 |
| 6 | CYS | HN | 5 | LYS+ | HB3 | 4.03 |
| 6 | CYS | HN | 6 | CYS | HA | 2.89 |
| 6 | CYS | HN | 6 | CYS | HB2 | 4.63 |
| 6 | CYS | HN | 6 | CYS | HB3 | 3.77 |
| 6 | CYS | HN | 7 | PHE | HN | 4.79 |
| 7 | PHE | QB | 7 | PHE | HA | 3.12 |
| 7 | PHE | QD | 7 | PHE | QB | 3.87 |
| 7 | PHE | QD | 9 | TYR | HA | 4.47 |
| 7 | PHE | QD | 9 | TYR | QE | 2.21 |
| 7 | PHE | QE | 7 | PHE | QD | 2.63 |
| 7 | PHE | HN | 6 | CYS | HB2 | 3.62 |
| 7 | PHE | HN | 6 | CYS | HB3 | 3.96 |
| 7 | PHE | HN | 7 | PHE | HA | 3.97 |
| 7 | PHE | HN | 7 | PHE | QB | 3.42 |
| 7 | PHE | HN | 14 | LEU | QD2 | 4.48 |
| 7 | PHE | HN | 15 | ALA | HB | 4.30 |
| 8 | ARG+ | HB2 | 5 | LYS+ | HN | 4.21 |
| 8 | ARG+ | HB2 | 8 | ARG+ | HA | 3.26 |
| 8 | ARG+ | HB2 | 8 | ARG+ | HD2 | 2.96 |
| 8 | ARG+ | HB2 | 8 | ARG+ | HN | 3.49 |
| 8 | ARG+ | HB3 | 8 | ARG+ | HA | 3.78 |
| 8 | ARG+ | HB3 | 8 | ARG+ | HD2 | 3.43 |
| 8 | ARG+ | HB3 | 8 | ARG+ | HG2 | 2.73 |
| 8 | ARG+ | HB3 | 8 | ARG+ | HN | 4.06 |
| 8 | ARG+ | HD2 | 8 | ARG+ | HG2 | 3.61 |
| 8 | ARG+ | HD3 | 5 | LYS+ | HN | 4.32 |
| 8 | ARG+ | HG2 | 8 | ARG+ | HA | 3.59 |
| 8 | ARG+ | HG2 | 8 | ARG+ | HD3 | 3.27 |
| 8 | ARG+ | HN | 7 | PHE | QB | 3.42 |
| 8 | ARG+ | HN | 7 | PHE | HN | 4.12 |
| 8 | ARG+ | HN | 8 | ARG+ | HA | 3.75 |
| 8 | ARG+ | HN | 8 | ARG+ | HG2 | 5.11 |
| 8 | ARG+ | HN | 8 | ARG+ | HG3 | 3.56 |
| 8 | ARG+ | HN | 9 | TYR | HN | 4.33 |
| 9 | TYR | HB2 | 9 | TYR | HA | 3.97 |
| 9 | TYR | HB3 | 10 | TYR | QD | 2.71 |
| 9 | TYR | QD | 9 | TYR | HA | 4.04 |
| 9 | TYR | QD | 9 | TYR | HB2 | 3.37 |
| 9 | TYR | QD | 9 | TYR | HB3 | 3.75 |
| 9 | TYR | QD | 9 | TYR | HN | 4.05 |
| 9 | TYR | QD | 13 | VAL | HB | 4.03 |
| 9 | TYR | QD | 13 | VAL | QG1 | 4.05 |
| 9 | TYR | QD | 15 | ALA | HB | 4.31 |
| 9 | TYR | QE | 7 | PHE | HA | 4.81 |
| 9 | TYR | QE | 7 | PHE | QB | 2.89 |
| 9 | TYR | QE | 7 | PHE | HN | 4.44 |
| 9 | TYR | QE | 8 | ARG+ | HN | 4.34 |
| 9 | TYR | QE | 9 | TYR | HA | 4.81 |
| 9 | TYR | QE | 15 | ALA | HB | 3.68 |
| 9 | TYR | QE | 17 | THR | HN | 5.12 |
| 9 | TYR | HN | 8 | ARG+ | HA | 2.69 |
| 9 | TYR | HN | 8 | ARG+ | HB3 | 4.31 |
| 9 | TYR | HN | 8 | ARG+ | HG2 | 4.59 |
| 9 | TYR | HN | 9 | TYR | HA | 3.87 |
| 9 | TYR | HN | 9 | TYR | HB2 | 2.97 |
| 9 | TYR | HN | 9 | TYR | HB3 | 3.79 |
| 9 | TYR | HN | 10 | PRO | QD | 4.97 |
| 9 | TYR | HN | 12 | GLY | HA2 | 4.54 |
| 9 | TYR | HN | 13 | VAL | HB | 4.26 |
| 9 | TYR | HN | 15 | ALA | HB | 5.13 |
| 10 | PRO | HB2 | 10 | PRO | HB3 | 3.19 |
| 10 | PRO | HB2 | 10 | PRO | QD | 2.97 |
| 10 | PRO | HB3 | 10 | PRO | HA | 2.33 |
| 10 | PRO | QD | 9 | TYR | HA | 2.55 |
| 10 | PRO | QD | 9 | TYR | HB2 | 3.83 |
| 10 | PRO | QD | 10 | PRO | HB3 | 3.86 |
| 10 | PRO | QD | 10 | PRO | HG2 | 3.61 |
| 10 | PRO | HG2 | 10 | PRO | HA | 3.85 |
| 10 | PRO | HG2 | 10 | PRO | HB2 | 2.33 |
| 10 | PRO | HG2 | 10 | PRO | HB3 | 2.65 |
| 10 | PRO | HG3 | 10 | PRO | HA | 3.00 |
| 10 | PRO | HG3 | 10 | PRO | HB2 | 3.00 |
| 10 | PRO | HG3 | 10 | PRO | HB3 | 3.12 |
| 10 | PRO | HG3 | 10 | PRO | QD | 3.01 |
| 10 | PRO | HG3 | 10 | PRO | HG2 | 2.29 |
| 11 | ASN | HB2 | 11 | ASN | HA | 2.81 |
| 11 | ASN | HB3 | 11 | ASN | HA | 2.67 |
| 11 | ASN | HD21 | 11 | ASN | HB2 | 3.73 |
| 11 | ASN | HD21 | 11 | ASN | HB3 | 3.66 |
| 11 | ASN | HD21 | 11 | ASN | HD22 | 2.07 |
| 11 | ASN | HD21 | 13 | VAL | QG2 | 4.02 |
| 11 | ASN | HD22 | 9 | TYR | HB2 | 4.18 |
| 11 | ASN | HD22 | 11 | ASN | HB2 | 3.94 |
| 11 | ASN | HD22 | 11 | ASN | HB3 | 3.95 |
| 11 | ASN | HN | 9 | TYR | HB2 | 3.87 |
| 11 | ASN | HN | 9 | TYR | HB3 | 3.68 |
| 11 | ASN | HN | 10 | PRO | HA | 3.87 |
| 11 | ASN | HN | 10 | PRO | HB3 | 3.74 |
| 11 | ASN | HN | 10 | PRO | QD | 4.21 |
| 11 | ASN | HN | 10 | PRO | HG2 | 4.13 |
| 11 | ASN | HN | 11 | ASN | HA | 4.36 |
| 11 | ASN | HN | 11 | ASN | HB2 | 4.71 |
| 11 | ASN | HN | 11 | ASN | HB3 | 3.74 |
| 11 | ASN | HN | 11 | ASN | HD21 | 3.01 |
| 11 | ASN | HN | 11 | ASN | HD22 | 4.99 |
| 11 | ASN | HN | 12 | GLY | HN | 3.74 |
| 11 | ASN | HN | 13 | VAL | QG2 | 4.84 |
| 12 | GLY | HN | 9 | TYR | HB2 | 3.57 |
| 12 | GLY | HN | 9 | TYR | HB3 | 5.28 |
| 12 | GLY | HN | 9 | TYR | HN | 4.03 |
| 12 | GLY | HN | 11 | TYR | HA | 3.53 |
| 12 | GLY | HN | 12 | GLY | HA2 | 3.48 |
| 12 | GLY | HN | 12 | GLY | HA3 | 3.04 |
| 12 | GLY | HN | 13 | VAL | HN | 3.46 |
| 13 | VAL | HA | 13 | VAL | HB | 4.94 |
| 13 | VAL | HA | 13 | VAL | QG2 | 2.86 |
| 13 | VAL | HA | 14 | LEU | HN | 2.59 |
| 13 | VAL | HB | 9 | VAL | HB2 | 2.82 |
| 13 | VAL | HB | 9 | VAL | HB3 | 3.66 |
| 13 | VAL | HB | 13 | VAL | QG1 | 2.64 |
| 13 | VAL | QG2 | 11 | ASN | HD22 | 3.97 |
| 13 | VAL | QG2 | 13 | VAL | HB | 2.82 |
| 13 | VAL | QG2 | 14 | LEU | HN | 4.67 |
| 13 | VAL | HN | 9 | VAL | HB3 | 4.78 |
| 13 | VAL | HN | 9 | VAL | HB2 | 3.83 |
| 13 | VAL | HN | 9 | VAL | HN | 4.59 |
| 13 | VAL | HN | 12 | GLY | HA2 | 3.97 |
| 13 | VAL | HN | 12 | GLY | HA3 | 3.88 |
| 13 | VAL | HN | 13 | VAL | HA | 3.60 |
| 13 | VAL | HN | 13 | VAL | HB | 3.08 |
| 13 | VAL | HN | 13 | VAL | QG2 | 3.27 |
| 13 | VAL | HN | 14 | LEU | HN | 4.74 |
| 14 | LEU | HB2 | 14 | LEU | HN | 3.09 |
| 14 | LEU | HB3 | 14 | LEU | HB2 | 2.21 |
| 14 | LEU | HB3 | 14 | LEU | QD1 | 3.65 |
| 14 | LEU | HB3 | 14 | LEU | QD2 | 3.48 |
| 14 | LEU | HB3 | 14 | LEU | HN | 3.24 |
| 14 | LEU | QD2 | 6 | CYS | HB2 | 3.80 |
| 14 | LEU | HG | 14 | LEU | QD1 | 3.55 |
| 14 | LEU | HG | 14 | LEU | QD2 | 3.04 |
| 14 | LEU | HN | 13 | VAL | HB | 4.40 |
| 14 | LEU | HN | 13 | VAL | QG1 | 3.47 |
| 14 | LEU | HN | 14 | LEU | QD2 | 5.26 |
| 14 | LEU | HN | 15 | ALA | HN | 4.40 |
| 15 | ALA | HB | 7 | PHE | QB | 3.91 |
| 15 | ALA | HB | 13 | VAL | QG1 | 3.58 |
| 15 | ALA | HB | 15 | ALA | HA | 3.21 |
| 15 | ALA | HB | 17 | THR | HN | 4.68 |
| 15 | ALA | HN | 7 | PHE | HN | 3.92 |
| 15 | ALA | HN | 8 | ARG+ | HA | 4.22 |
| 15 | ALA | HN | 9 | TYR | HN | 4.55 |
| 15 | ALA | HN | 13 | VAL | QG1 | 4.85 |
| 15 | ALA | HN | 14 | LEU | HA | 2.62 |
| 15 | ALA | HN | 14 | LEU | HB3 | 4.71 |
| 15 | ALA | HN | 14 | LEU | QD2 | 4.08 |
| 15 | ALA | HN | 14 | LEU | HG | 4.33 |
| 15 | ALA | HN | 15 | ALA | HA | 3.93 |
| 15 | ALA | HN | 15 | ALA | HB | 3.42 |
| 16 | CYS | HA | 17 | THR | HN | 2.84 |
| 16 | CYS | HN | 15 | ALA | HA | 3.02 |
| 16 | CYS | HN | 15 | ALA | HB | 4.03 |
| 16 | CYS | HN | 16 | CYS | HA | 4.20 |
| 16 | CYS | HN | 16 | CYS | QB | 3.24 |
| 17 | THR | HN | 17 | THR | HA | 4.10 |
| 17 | THR | HN | 17 | THR | HB | 3.90 |
| 17 | THR | HN | 17 | THR | QG2 | 4.54 |
