# Supplementary material for: Odorranalectin Is a Small Peptide Lectin with Potential for Drug Delivery and Targeting
Source: PLoS One. 2008 Jun 11;3(6):e2381. doi: 10.1371/journal.pone.0002381 (PMC2440032; doi:10.1371/journal.pone.0002381)
Supplement: Table S9 — 3J (HN-HA) scalar coupling constants (Hz) used for structure calculation of odorranalectin as backbone dihedral angle restraints (0.03 MB DOC) [file pone.0002381.s013.doc]

Table S9. ^3^J (HN-HA) scalar coupling constants (Hz) used for structure calculation

of odorranalectin as backbone dihedral angle restraints.

| Residue number | Residue name | J-couplings |
| --- | --- | --- |
| 3 | SER | 5.4 |
| 5 | LYS | 8.4 |
| 6 | CYS | 8.4 |
| 7 | PHE | 8.4 |
| 8 | ARG | 8.4 |
| 9 | TYR | 6.6 |
| 11 | ASN | 6.6 |
| 13 | VAL | 5.4 |
| 14 | LEU | 8.4 |
| 15 | ALA | 8.4 |
| 16 | CYS | 9.0 |
| 17 | THR | 8.4 |
