# Supplementary material for: Odorranalectin Is a Small Peptide Lectin with Potential for Drug Delivery and Targeting
Source: PLoS One. 2008 Jun 11;3(6):e2381. doi: 10.1371/journal.pone.0002381 (PMC2440032; doi:10.1371/journal.pone.0002381)
Supplement: Table S10 — 1H chemical shifts of odorranalectin in H2O at 298 K (0.03 MB DOC) [file pone.0002381.s014.doc]

Table S10 ^1^H chemical shifts of odorranalectin in H_2_O at 298 K.

__________________________________________________________________________________

Residue NH H_α_ H_β_ H_γ_ others

Tyr1 7.607, 6.934

Ser3 8.324 4.706 3.847, 3.896

Pro4 4.484 2.028, 2.282 1.903, 2.037 3.736, 3.862

Lys5 8.532 4.413 1.788, 1.876 1.399, 1.468 1.691, 2.987

Cys6 8.312 5.430 2.725, 3.065

Phe7 8.798 4.616 2.930 6.974, 7.146

Arg8 8.131 4.868 1.511, 1.622 1.381, 1.514 3.091, 3.111

Tyr9 8.861 4.671 3.120, 3.259 6.866, 7.291

Pro10 4.405 2.227, 2.460 1.996, 2.126 4.048

Asn11 7.796 4.586 2.876, 3.210 6.563, 7.412

Gly12 8.523 3.626, 4.304

Val13 7.542 3.864 2.146 0.910, 0.941

Leu14 8.413 4.774 1.439, 1.707 1.491 0.765, 0.814

Ala15 8.929 4.683 1.253

Cys16 8.689 5.429 3.027

Thr17 8.320 4.314 4.237 1.244
