# Supplementary material for: Odorranalectin Is a Small Peptide Lectin with Potential for Drug Delivery and Targeting
Source: PLoS One. 2008 Jun 11;3(6):e2381. doi: 10.1371/journal.pone.0002381 (PMC2440032; doi:10.1371/journal.pone.0002381)
Supplement: Table S11 — Structure statistics from NMR structure analysis (0.04 MB DOC) [file pone.0002381.s015.doc]

Table S11 Structure statistics from NMR structure analysis

| Quantity Value | | |
| --- | --- | --- |
| Total unambiguous distance restraints  Intra residual  Sequential ( | i – j | = 1)  Medium (2 ≤|I – j|≤ 4)  Long range ( | i – j | ≥ 5) | 187 | |
|  | 101 | |
|  | 45 | |
|  | 29 | |
|  | 12 | |
| RMSD from the average atomic coordinates （residues 4-16, Å） | | |
| Backbone atoms  All heavy atoms | 0.54 ± 0.16 | |
|  | 0.95 ± 0.23 | |
| Deviations from idealized covalent geometry | | |
| Bond (Å)  Angles (°)  Improper (°) | | 0.004 ± 0.0002 |
|  |  | 0.54 ± 0.03 |
|  |  | 1.51 ± 0.17 |
| Ramachandran analysis (%) | | |
| Residues in most favored regions  Residues in additional allowed regions  Residues in generously allowed regions  Residues in disallowed regions | 60.0% | |
|  | 35.4% | |
|  | 4.6% | |
|  | 0.0% | |
